# A shared mechanism of muscle wasting in cancer and Huntington’s disease

**DOI:** 10.1186/s40169-015-0076-z

**Published:** 2015-12-14

**Authors:** Michal Mielcarek, Mark Isalan

**Affiliations:** Department of Life Sciences, Imperial College London, Sir Alexander Fleming Building (SAF), London, SW7 2AZ UK

**Keywords:** Skeletal muscle atrophy, Muscle cachexia, Huntington’s disease, Neurodegeneration, Cancer, Energy imbalance

## Abstract

Skeletal muscle loss and dysfunction in aging and chronic diseases is one of the major causes of mortality in patients, and is relevant for a wide variety of diseases such as neurodegeneration and cancer. Muscle loss is accompanied by changes in gene expression and metabolism that lead to contractile impairment and likely affect whole-body metabolism and function. The changes may be caused by inactivity, inflammation, age-related factors or unbalanced nutrition. Although links with skeletal muscle loss have been found in diseases with disparate aetiologies, for example both in Huntington’s disease (HD) and cancer cachexia, the outcome is a similar impairment and mortality. This short commentary aims to summarize recent achievements in the identification of common mechanisms leading to the skeletal muscle wasting syndrome seen in diseases as different as cancer and HD. The latter is the most common hereditary neurodegenerative disorder and muscle wasting is an important component of its pathology. In addition, possible therapeutic strategies for anti-cachectic treatment will be also discussed in the light of their translation into possible therapeutic approaches for HD.

## Background

Huntington’s disease (HD) is the most common monogenic neurodegenerative disorder, affecting approximately 1 in 10,000 people worldwide [1]. It is invariably fatal and there is no approved treatment that targets the molecular cause of the disease. This disorder has been primarily characterized by choreiform movements, psychiatric symptoms and slowly progressive dementia. Consequently, brain pathology has been recognised as the major hallmark of HD; for a review see [2]. On the molecular level, HD is caused by the expansion of a polyglutamine stretch within the huntingtin protein (HTT). This mutation leads to an extra-long tract of glutamines within the HTT that causes the huntingtin protein to aggregate [3]. The genetic mutation within the huntingtin locus (HTT) leads to a widespread neurodegeneration, particularly in the striatal nuclei, basal ganglia and cerebral cortex in humans.

Importantly, although HD is widely thought of as solely a neurological disease, recent studies have emphasized detrimental pathologies that occur within peripheral tissues, identifying them as an important component of HD pathogenesis. Peripheral pathologies include HD-related cardiomyopathy [4–6] or skeletal muscle malfunction; for a review see [7]. Muscle malfunction is not only a well-documented symptom of HD but is also apparent for other neurodegenerative disorders such as spinal cerebellar ataxia-17 (SCA17) [8], Alzheimer’s disease [9] and in a mouse model of stroke [10]. Therefore, despite the fact that HD is still recognised principally as a neurological disease, peripheral pathologies including skeletal muscle malfunctions might significantly contribute to the overall progression of HD.

### A shared mechanism of muscle syndrome in HD and cancer cachexia

Muscle wasting syndrome is a well-documented symptom, manifested by molecular and physiological changes that can be detected even in pre-symptomatic HD individuals; for a review see [7]. Our recent study clearly identified a progressive skeletal muscle atrophy, demonstrated by mass decline in all type skeletal muscles, in two well-characterised and widely studied mouse models of HD: R6/2 and *Hdh*Q150 [11]. Similarly, the C26 cachectic mouse model [mice bearing colon-26 (C-26) tumors] developed an atrophy of both glycolytic and oxidative fibres [12], followed by a decline in grip strength and rotarod performance [13]. In HD models, this was accompanied by the contractile dysfunction of the hind limb muscles, tibialis anterior (TA) and extensor digitorum longus (EDL), followed by a significant loss of motor units. In addition, these functional muscle impairments were accompanied by an aberrant deregulation of contractile protein transcripts and their up-stream transcriptional regulators [11]. There was also a significant reduction in muscle force, likely due to an energy metabolism imbalance and decreased oxidation, in both fast and slow types of skeletal muscle fibres [11]. In general, it is believed that mitochondrial dysfunction and energy deficits underline HD pathology; for a review see [14]. Our results were in line with a previous study in the R6/2 mouse model that showed increased levels of circulating markers of muscle injury in the serum and a reduction of contractile transcripts [15]. It is interesting to compare these results to those found in cancer cachexia; animal models of urothelial carcinoma or Lewis lung carcinoma showed decreased respiratory chain activity [16] and a lower level of ATP [17], respectively, similarly to HD mouse models [11].

Interestingly, HD-related skeletal muscle syndrome has been directly correlated to the malfunction of the histone deacetylase 4 (HDAC4)–dachshund homolog 2 (DACH2)–myogenin axis and is thus linked to transcriptional dysregulation. HDAC4 has already been identified as a molecular target of muscle dysfunction and has been linked to disease progression in amyotrophic lateral sclerosis (ALS) [18, 19] and spinal muscular atrophy (SMA) [20]. There is also strong evidence that the genetic reduction of HDAC4 in skeletal muscle can contribute to an overall improvement of HD phenotypes [21, 22]. Although HDAC4 function in muscle remodelling has been well-established in various models of neurodegenerative disorders (see [23] for a review), there is no evidence so far that HDAC4 can play a similar detrimental function in cancer cachexia. On the other hand, an elegant study clearly identified the HDAC4-activated [24] transcription factor paired box 7 (Pax-7) as sufficient for inducing atrophy in normal muscle [25]. Furthermore, the reduction of Pax7, or the exogenous addition of its downstream targets, like MyoD, reversed muscle wasting by restoring cell differentiation and fusion, under tumor conditions. Interestingly, Pax7 was induced by serum factors from cachectic mice or patients, in an Nuclear Factor kappa B (NFκB)-dependent manner, both in vitro and in vivo. Together, these data suggest that circulating cachectic factors induce muscle damage and activation of satellite cells at an early stage of cachexia development, by perturbing transcription networks [25]. However, such pathways have not been validated in HD mouse models thus far.

In fact, the source of skeletal muscle wasting in HD has not yet been identified. On the one hand, wide-spread neurodegeneration including that of the hypothalamus could be a cause of muscle malfunction. However, it is also possible that an intrinsic component of the HTT mutant gene product, expressed within muscle cells, might directly lead to pathogenic consequences. In addition, it has been shown that R6/2 mice had elevated levels of NFκB pathways that may be involved in muscle atrophy [15]. Similarly, increased levels of pro-inflammatory cytokines like tumor necrosis factors (TNF) and interleukin 1 (IL-1), caused by dysfunction of hypothalamic serotonergic neurons, have been implicated in cancer cachexia [26]. It has been clearly demonstrated that injection of IL-1 into the hypothalamus causes a significant change in gene expression in skeletal muscle within hours, leading to their degradation [27]. Thus, it is likely that a combination of dysregulated cytokines and transcription regulators come together to achieve the common result of muscle wasting, in both HD and cancer cachexia.

## Conclusions

### Therapeutic strategies for targeting muscle wasting

It is becoming more widely accepted that therapeutic approaches in HD should not only be restricted to targeting the brain pathology but also major efforts should be made to understand the related peripheral pathologies, including those of skeletal muscles. Here, major insights may be gained by examining research in the field of cancer cachexia.

In cancer cachexia, the most promising therapeutic approach is based on the inhibition of the myostatin pathway to rescue muscle loss. Myostatin promotes skeletal muscle wasting in different catabolic conditions, including cancer [28] and it has been shown to be secreted by cancer cells [29]. In fact, inactivation of myostatin by treatment with a soluble form of activin receptor IIB (sACTRIIB) ablated the symptoms of cancer cachexia in mice bearing Lewis lung carcinoma [30]. The other promising therapeutic approach is based on the activity of the transcription factor phospho-signal transducer and activator of transcription 3 (p-Stat3) and, indeed, its inhibition with a small molecule chemical led to improvements of muscle mass losses, increased body weight and grip strength in both Lewis lung carcinoma and C-26 tumor mouse models [31]. Muscle atrophy has been also linked to the forkhead box O3 (Foxo-3) transcription factor and its overexpression in skeletal muscle was sufficient to induce dramatic skeletal muscle wasting [32]. Conversely, inhibition of Foxo genes spared muscle loss in a mouse model of cancer cachexia [33].

**Fig. 1:**
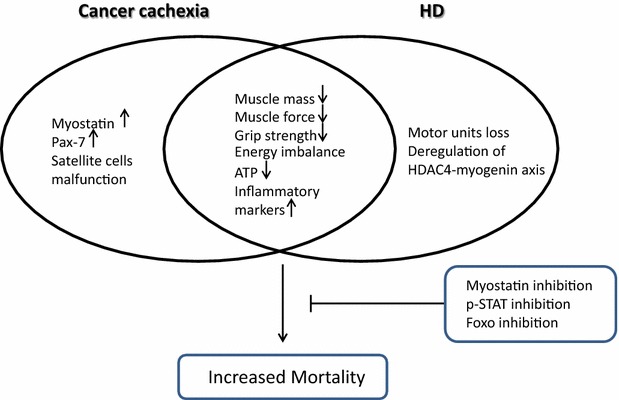
A summary of key common pathological features in skeletal muscle wasting syndrome comparing cancer cachexia and Huntington’s disease (HD), based on the published literature [11–13, 15–17, 25, 28]. The *left portion* presents specific molecules and pathways that have been identified only in cancer cachexia models, but not explored in HD, while the *right portion* presents unique pathological features of HD-related skeletal muscle pathology. The *intersection* summarizes shared features of muscle pathology in HD and cancer cachexia. The *box* (*bottom right*) summarizes effective therapeutic approaches to prevent muscle wasting, in pre-clinical settings of cancer cachexia [30, 31, 33], that could be beneficial in HD

Based on the above, it will be vital to dissect whether promising therapeutic approaches in the cancer cachexia mouse models could be beneficial in HD, as they may well share common features of muscle wasting syndrome (Fig. 1). Despite these common features, it will still be vital to understand the specific mechanisms leading to HD-related striated muscle pathology, in pre-clinical and clinical settings. For example, HDAC4 reduction expands the life span of the very aggressive R6/2 HD mouse model by approximately 20 %, and clearly offers a promising alternative therapeutic target which needs to be further characterised, not only in the CNS but also in skeletal muscle [21, 22], since HDAC4 has also been linked to muscle ageing in humans [34]. By considering the role of muscle wasting in hitherto unconnected diseases, it will likely be possible to shape future therapeutic strategies for a wide range of pathologies.

## Abbreviations

HD: Huntington’s disease
TA: tibialis anterior
EDL: extensor digitorum longus
ACTRIIB: activin receptor type IIB
ALS: amyotrophic lateral sclerosis
SMA: spinal muscular atrophy
NFκB: nuclear factor kappaB
HDAC4: histone deacetylase 4
DACH2: dachshund homolog 2
Pax7: paired box 7
IL-1: interleukin 1
p-Stat3: phospho-signal transducer and activator of transcription 3
FOXO-3: forkhead box O3
